# Efficacy and Moderators of Cognitive Behavioural Therapy for Psychosis Versus Other Psychological Interventions: An Individual-Participant Data Meta-Analysis

**DOI:** 10.3389/fpsyt.2020.00402

**Published:** 2020-05-05

**Authors:** David T. Turner, Mirjam Reijnders, Mark van der Gaag, Eirini Karyotaki, Lucia R. Valmaggia, Steffen Moritz, Tania Lecomte, Douglas Turkington, Rafael Penadés, Helio Elkis, Corinne Cather, Frances Shawyer, Kieron O’Connor, Zhan-Jiang Li, Eliza Martha de Paiva Barretto, Pim Cuijpers

**Affiliations:** ^1^Department of Clinical, Neuro and Developmental Psychology, Amsterdam Public Health Research Institute, Vrije Universiteit, Amsterdam, Netherlands; ^2^Parnassia Psychiatric Institute, The Hague, Netherlands; ^3^Department of Psychology, Institute of Psychiatry, Psychology and Neuroscience, King’s College London, London, United Kingdom; ^4^South London and Maudsley NHS Trust, London, United Kingdom; ^5^Klinik für Psychiatrie und Psychotherapie, Arbeitsgruppe Klinische Neuropsychologie, Universitätsklinikum Hamburg-Eppendorf, Hamburg, Germany; ^6^Département de Psychologie, Université de Montréal, Montréal, QC, Canada; ^7^Wolfson Unit, Centre for Aging and Vitality, Newcastle-upon-Tyne, United Kingdom; ^8^Hospital Clínic Barcelona, University of Barcelona, IDIBAPS-CIBERSAM, Barcelona, Spain; ^9^Department and Institute of Psychiatry, University de São Paulo Medical School, São Paulo, Brazil; ^10^Massachusetts General Hospital and Harvard Medical School, Boston, MA, United States; ^11^Southern Synergy, Department of Psychiatry, Faculty of Medicine, Nursing and Health Sciences, School of Clinical Sciences at Monash Health, Monash University, Melbourne, VIC, Australia; ^12^Department Psychiatrie, Université de Montréal, Montréal, QC, Canada; ^13^Department of Clinical Psychology, Beijing Anding Hospital, Capital Medical University, Beijing, China

**Keywords:** psychosis, cognitive–behavioural therapy, individual-participant data, meta-analysis, psychological intervention

## Abstract

**Background:**

Study-level meta-analyses have demonstrated the efficacy of cognitive–behavioural therapy for psychosis (CBTp). Limitations of conventional meta-analysis may be addressed using individual-participant-data (IPD). We aimed to determine a) whether results from IPD were consistent with study-level meta-analyses and b) whether demographic and clinical characteristics moderate treatment outcome.

**Methods:**

We systematically searched PubMed, Embase, PsychInfo and CENTRAL. Authors of RCTs comparing CBTp with other psychological interventions were contacted to obtain original databases. Hierarchical mixed effects models were used to examine efficacy for psychotic symptoms. Patient characteristics were investigated as moderators of symptoms at post-treatment. Sensitivity analyses were conducted for risk of bias, treatment format and study characteristics.

**Results:**

We included 14 of 23 eligible RCTs in IPD meta-analyses including 898 patients. Ten RCTs minimised risk of bias. There was no significant difference in efficacy between RCTs providing IPD and those not (*p >*0.05). CBTp was superior vs. other interventions for total psychotic symptoms and PANSS general symptoms. No demographic or clinical characteristics were robustly demonstrated as moderators of positive, negative, general or total psychotic symptoms at post-treatment. Sensitivity analyses demonstrated that number of sessions moderated the impact of treatment assignment (CBTp or other therapies) on total psychotic symptoms (*p* = 0.02).

**Conclusions:**

IPD suggest that patient characteristics, including severity of psychotic symptoms, do not significantly influence treatment outcome in psychological interventions for psychosis while investing in sufficient dosage of CBTp is important. IPD provide roughly equivalent efficacy estimates to study-level data although significant benefit was not replicated for positive symptoms. We encourage authors to ensure IPD is accessible for future research.

## Introduction

The efficacy of psychological interventions for psychosis have been established (1–5) while counter-argument questioning *effectiveness* exists (6, 7). Meta-analytic studies represent the pinnacle of evidence-based psychological intervention in psychosis. Using traditional “two-step” study-level meta-analytic methods in pooling effect sizes from published articles, we have demonstrated that cognitive–behavioural therapy for psychosis (CBTp) represents the most efficacious psychological intervention for positive symptoms in psychosis (8), while social skills training is most efficacious in the treatment of negative and general symptoms (9).

There are however inherent limitations of the conventional “two-step” approach. Comparisons often lack adequate power to detect effects hence risk Type II errors, while precision of effect size estimates may be improved. Lack of power and poor availability of relevant variables at the study-level also preclude identification of moderators of treatment outcome (10). Individual-participant data (IPD) meta-analyses address these issues by utilising original databases from RCTs rather than relying on data from published trials. This approach maximises power to detect effects and allows the examination of moderators *via* participant characteristics that vary at the IPD level (11).

IPD methodology has been applied to psychosis research, including investigation of non-response rates to antipsychotic medication (12). We note that IPD meta-analysis is distinct to network meta-analysis and cumulative meta-analysis, two other novel meta-analytic methods that have recently been applied in psychosis-related research (13–15). The present meta-analysis is, to our knowledge, the first attempt to apply IPD methodology to psychological interventions in psychosis. We report the results of an IPD meta-analysis comparing CBTp to other psychological interventions alongside an exploratory moderator analysis investigating the impact of demographic and clinical characteristics on treatment outcome. We had two research objectives; 1) to determine whether evidence for the efficacy of CBTp from IPD is consistent with previous meta-analytic evidence and 2) to determine whether demographic and clinical characteristics of psychosis patients moderate the outcome of psychological therapies. We hypothesised that IPD would provide broadly equivalent efficacy outcomes to previous research while our moderator analysis was conducted in an exploratory manner based upon available IPD without pre-specified hypotheses.

## Methods

### Identification and Inclusion of Studies

A systematic literature search was completed on the 25th September 2017. The search strategy has been described elsewhere (8) and is included in the **Supplementary Materials**. We examined 7,037 abstracts from four databases: Pubmed (2,011), PsycInfo (2,457), Embase (1,071) and the Cochrane Central Register of Controlled Trials (1,498). Abstracts were identified by combining terms indicative of psychological interventions for psychosis and relevant psychotic disorders (MeSH terms and text words). We checked reference lists from earlier meta-analyses to ensure that no published studies were missed. From 7,037 abstracts (5,881 after the removal of duplicates), we retrieved 621 full-text papers for consideration.

We included (a) RCTs in which (b) CBTp (c) was compared with another psychological intervention (d) for patients with a psychotic disorder, (e) based on an established standardised diagnostic interview, (f) in which the aim was to reduce psychotic or psychiatric symptoms.

The psychological interventions that were included as comparison conditions are operationally defined elsewhere (8). Studies targeting patients with comorbid general medical disorders or prodromal psychosis were excluded. Trials were excluded if the comparison condition was not an active psychological intervention (e.g. treatment as usual, waiting list). Medication adherence or compliance RCTs was excluded. Language restrictions were set to English and German.

After identifying potential RCTs for inclusion, the corresponding authors of each were contacted by email and invited to participate by providing the sociodemographic and clinical characteristics alongside the outcome data from their trials. If authors did not respond within two weeks a reminder was sent. If no answer was received, we considered the trial unavailable. In instances in which authors responded but were unsure whether data could be provided, contact was maintained until it was clear that data was unobtainable.

### Risk of Bias Assessment

The risk of bias of the included RCTs was assessed using four criteria of the Cochrane Collaboration risk of bias tool (16); sequence generation, allocation concealment, blinding of outcome assessors and incomplete outcome data. Only the data reported in the published papers was used as this was considered to be the most conservative estimate. Two independent researchers (DT and EK) carried out the risk of bias assessment. Disagreements were resolved through discussion.

### Assessment of Psychotic Symptoms

Psychotic symptoms were measured using three commonly used scales measuring positive, negative and general symptoms of psychosis; the Positive and Negative Syndrome Scale [PANSS. (14)], the Brief Psychiatric Rating Scale [BPRS. (15, 16)] and the Scale for the Assessment of Negative Symptoms [SANS. (17)]. Further information on these scales is provided in the **Supplementary Materials**. In instances of multi-scale use, we selected the main outcome using the following rank order: (1) PANSS; (2) BPRS; (3) SANS. To facilitate comparison across RCTs and outcome measures, a standardised variable was created each for the combined positive, negative and total subscales using z-scores. Total and subscale scores per participant were utilised rather than item-level data therefore we relied on scoring algorithms applied in the original RCTs. Higher scores indicated greater severity in all scales.

### Differences Between Included and Non-Included RCTs

To examine whether RCTs included in the IPD meta-analysis differed in post-treatment outcome from RCTs for which we were unable to obtain databases, we completed conventional “two step” meta-analyses. We obtained comparative effect sizes using Comprehensive Meta-Analysis software (CMA; version 2.2.057). We corrected for small samples based on the procedures suggested by Hedges and Olkin (18) therefore provided effect sizes in Hedge’s *g*.

### Publication Bias

Publication bias was tested in all RCTs meeting inclusion criteria and in the subset included in IPD meta-analyses. We inspected funnel plots and applied Duval and Tweedie’s trim and fill procedure (19). We also conducted Egger’s test of the intercept to quantify bias captured by the funnel plot and test for significance.

### Missing Data

Participants with missing baseline data were deleted from the IPD dataset (*n* = 12). The proportion of missing post-treatment outcome data was 9% (*n* = 80) for the PANSS and 3% (*n* = 26) for the other psychotic symptom scales. Missing outcome data at post-treatment was not imputed. It has been repeatedly demonstrated in IPD meta-analyses that imputed analyses do not significantly differ from completer analyses (10, 20, 21) while mixed models already make optimal use of available data.

### IPD Meta-Analyses

All analyses were conducted using the ‘xtmixed’ command in Stata/SE software (version 14.2). Firstly, we applied a mixed effects model to examine the efficacy of CBTp vs. other psychological interventions in reducing positive, negative, general, and total psychotic while controlling for baseline psychotic symptom severity and accounting for clustering of patients within studies. These analyses were conducted using all the separate standardised subscales of the PANSS (positive, negative, general, and total), the BPRS (positive, negative, and total), and the SANS (total) as dependent variables. The analyses were repeated using all standardised positive subscales combined, all standardised negative subscales, and all standardised total subscales as dependent variables. Both the treatment dummy (CBTp = 1 and other therapeutic interventions = 0), and psychotic symptom severity at baseline were used as predictors in the models.

We again used a mixed effects model to examine whether sociodemographic and clinical variables moderate the efficacy of CBTp vs. other psychological interventions in reducing positive, negative and total psychotic symptoms while controlling for baseline psychotic symptom severity and accounting for clustering of patients within studies. Sociodemographic moderator variables included age, gender, marital status (married; not married), education level (secondary/lesser; tertiary/further), ethnicity (Caucasian; ethnic minority), occupation (employed; unemployed; student), type of diagnosis (schizophrenia; schizo-affective disorder; other), and illness duration in years. Clinical moderator variables included the PANSS negative and general psychotic symptoms at baseline and number of treatment sessions. The treatment dummy, psychotic symptom severity at baseline and the interaction between the treatment dummy and the moderators were used as predictors. All analyses were carried out per moderator, using the combined standardised positive, negative and total subscales as dependent variables. All continuous moderator variables were centred on the study level, to ensure that the interaction term explains only patient level variation in treatment response instead of study level variation.

Finally, sensitivity analyses were conducted in which all of the previously described analyses were redone using only studies that were assessed as having minimal risk of bias. We also conducted post-hoc sensitivity analyses in instances where there were conceptual differences between included studies in interventions, outcomes and treatment format (group vs individual).

## Results

### Selection of Studies

**Figure 1:**
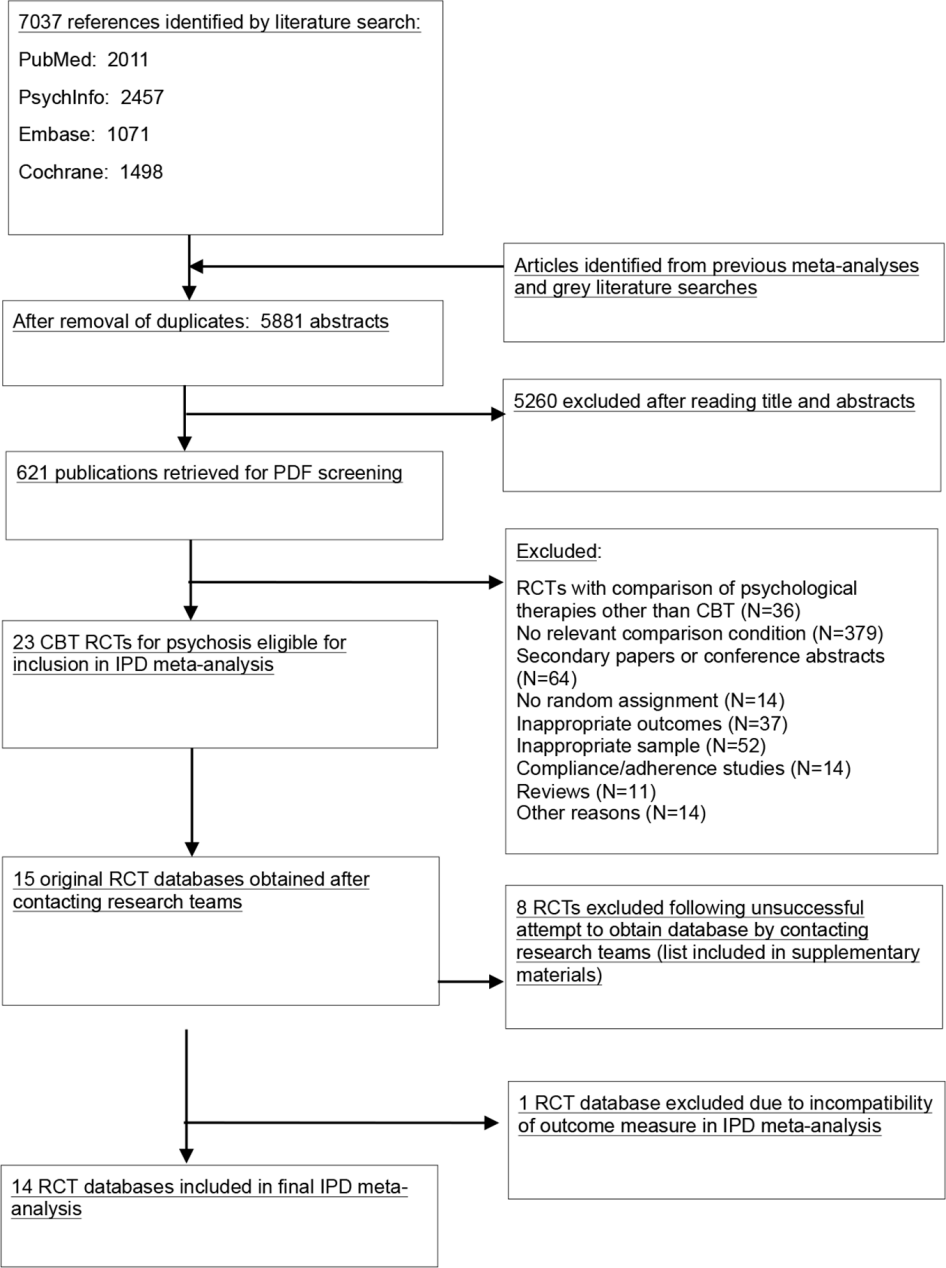
Flowchart of inclusion of studies.

**Figure 1** provides a flowchart describing the inclusion process. Of 621 full-text papers retrieved, 598 were excluded while 23 RCTs met our inclusion criteria. Of these 23 studies, 15 provided patient-level data (65%). Eight studies for which authors were contacted did not contribute data and were therefore excluded from the IPD meta-analysis (please see the **Supplementary Materials** for a list of these RCTs). One study did contribute (22) data but utilised an outcome measure which was not comparable to other RCTs and was therefore excluded. This resulted in 14 trials being included in the IPD meta-analysis.

### Characteristics of Included Studies and Patients

Study characteristics are summarised in **Table 1**. The 14 RCTs included a total of 898 patients. 460 received CBTp and 438 received other psychological interventions. Comparison interventions were befriending (five RCTs), supportive counselling (4), cognitive remediation (2), socials skills training (1), psychoeducation (1) and family intervention (1). Four studies were conducted in the UK, two in the US, two in Canada, two in Australia, and one in China, Brazil, Germany, Spain and the Netherlands. Eleven utilised individual treatment format, two used group format and one implemented both. Treatment duration ranged from 4 to 52 weeks. A summary of patient characteristics is provided in the **Supplementary Materials** alongside a histogram summarising the distribution of PANSS total severity at baseline. The mean PANSS total score at baseline was 71, which falls within the *moderately ill* range (38) and is comparable to previous meta-analyses (39).

**Table 1:** Selected characteristics of randomised controlled trials of CBTp versus other psychological interventions for psychosis.

Study & publications	Country	Sample characteristics	Relevant comparisons & *n*	Symptom outcome measures	Format	Bias Risk (0-4)	Duration (weeks to PT)	Follow-up
Barretto et al. (23)	Brazil	DSM-IV Schizophrenia, 6 months clozapine treatment-resistant. Outpatients.	CBT (12) vs. BF (10)	BPRS, PANSS	Individual	2	21	6 months
Cather et al. (24)	USA	Schizophrenia or schizoaffective disorder. Outpatients	CBT (15) vs. PE (13)	PANSS, PSYRATS	Individual	1	16	N/A
Durham et al. (25)	UK	Schizophrenia, Schizoaffective disorder or delusional disorder suffering positive symptoms. Outpatient & inpatient.	CBT (22) vs. SC (23)	PANSS, PSYRATS	Individual	0	39	3 months
Garety et al. (26)	UK	Recently relapsed non-affective psychosis (ICD 10 F2 & DSM-IV), with carers. Positive symptoms.	CBT (27) vs. FI (28)	PANSS, PSYRATS,	Individual	0	52	24 months
Haddock et al. (27)	UK	DSM-IV schizophrenia or schizoaffective disorder. History of violence. Current anti-psychotic medication & positive symptoms.	CBT (38) vs. BF (39)	PANSS, PSYRATS	Individual	0	26	12 months
Jackson et al. (28)	Australia	First episode psychosis including schizophrenia, schizophreniform, schizoaffective, bipolar, delusional disorder & psychosis NOS. Inpatient & outpatient.	CBT (31) vs. BF (31)	BPRS, SANS	Individual	2	12	12 months
Lecomte et al. (29)	Canada	Early psychosis (< 2 years). Current psychotic symptoms. Stabilized outpatients.	CBT (48) vs. SST (54)	BPRS	Group	2	13	6 months, 12 months
Li et al. (30)	China	DSM-IV schizophrenia. Adequate antipsychotic dose. Inpatients & outpatients.	CBT (96) vs. SC (96)	PANSS	Individual	0	24	12, 36 & 60 weeks
Moritz et al. (31)	Germany	Broad psychotic inpatients meeting criteria for schizophrenifom disorder.	CBT (24) vs. CR (24)	PANSS, PSYRATS	Both	0	4	N/A
Penades et al. (32)	Spain	DSM-IV schizophrenia. Chronic. Prevalence of negative symptoms & cognitive impairment.	CBT (20) vs. CR (20)	PANSS	Individual	0	17	6 months
Penn et al. (33)	USA	Schizophrenia or schizoaffective disorder & current auditory hallucinations. Outpatients.	CBT (32) vs. SC (33)	PANNS, PSYRATS	Group	0	12	3 months, 12 months
Sensky et al. (34) & Turkington et al. (35)	UK	DSM-IV & ICD-10 schizophrenia. Treatment resistant. Outpatients.	CBT (46) vs. BF (44)	CPRS, SANS,	Individual	0	39	9 months, 5 years
Shawyer et al. (36)	Australia	DSM-IV schizophrenia or related condition including command hallucinations in previous 6 months. Outpatients.	CBT (21) vs. BF (22)	PANSS, PSYRATS, CH	Individual	0	15	6 months
Valmaggia et al. (37)	Netherlands	DSM-IV schizophrenia including residual delusions or auditory hallucinations. Medication resistant.	CBT (36) vs. SC (26)	PANSS, PSYRATS	Individual	0	22	6 months

BF, Befriending; BPRS, Brief Psychiatric Rating Scale; CBT, Cognitive–Behavioural Therapy; CH, Command Hallucinations; CPRS, Comprehensive Psychopathological Rating Scale; CR, Cognitive Remediation; FI, Family Intervention; n, Number of participants in each treatment group; PANSS, Positive and Negative Symptoms Scale; PE, Psycho-education; PSYRATS, Psychotic Symptom Rating Scale; PT, Post-treatment SANS, Scale for Assessment of Negative Symptoms; SC, Supportive Counselling; SST, Social Skills Training.

### Risk of Bias

Risk of bias varied between RCTs (**Table 1** and **Supplementary Materials**). Of the 14 studies, 10 reported adequate sequence generation and nine reported satisfactory allocation concealment. All studies reported blinding of outcome assessors. All studies utilised intention-to-treat analyses to address missing outcome data. 10 studies were assessed as successfully minimising all four risk of bias criteria, while four successfully met two or three criteria. No studies were assessed as having the highest possible risk of bias score.

### Available and Unavailable Data: Conventional Meta-Analysis

To test for differences between available and unavailable data, we ran a conventional meta-analysis comparing the 14 studies included in the IPD meta-analysis with the 9 trials which met our inclusion criteria but did not contribute primary data. For total symptoms with all 23 studies included, results showed a small significant effect in favour of CBTp (*g* = 0.16, *p* = 0.01). Analysing only the 14 studies included in the IPD meta-analysis resulted in a small significant effect in favour of CBTp (*g* = 0.17, *p* = 0.01). There was no significant effect when analysing the nine remaining non-included studies although the magnitude of effect size was similar (*g* = 0.14, *p* = 0.28). The difference between the IPD studies and those not-included was not significant (*p* = 0.80).

For positive symptoms it was possible to include 16 studies in the overall comparison; results demonstrated a small significant effect in favour of CBTp (*g* = 0.15, *p* = 0.03). Including only the 11 IPD studies resulted in a small non-significant effect in favour of CBTp (*g* = 0.13, *p* = 0.09). The effect was also non-significant when analysing the remaining 5 non-included studies (*g* = 0.19, *p* = 0.12). The difference between the IPD and non-IPD studies was not significant (*p* = 0.65). For negative symptoms, CBTp did not demonstrate significant superiority when all 10 available studies were included (*g* = 0.05, *p* = 0.52), nor when analysing only the 6 IPD studies (*g* = 0.06, *p* = 0.60) or the four remaining non-IPD studies (*g* = 0.04, *p* = 0.71). The difference between the IPD and non-IPD studies was not significant (*p* = 0.92).

### Publication Bias

The funnel plots assessing publication bias for the total symptoms and positive symptoms analyses on the overall 23 studies suggested the existence of one unpublished negative trial in each. Egger’s (40) test did not suggest that the extent of publication bias was significant for the total (*p* = 0.13) or positive (*p* = 0.10) symptoms comparisons. The classic fail-safe *N* estimated that it would require 32 and 15 missing trials respectively to cause loss of effect significance. Duval and Tweedie’s (19) trim and fill procedure trimmed one study in each comparison, resulting in a marginal reduction in the magnitude of effect in both total symptoms (*g* = 0.14, 95% *CI*: 0.03–0.23) and positive symptoms (*g* = 0.13, 95% *CI*: −0.00–0.24). This resulted in the positive symptoms comparison losing significance. There was no evidence of publication bias in the negative symptoms comparison.

### IPD Meta-Analyses

#### Baseline Differences

We tested for differences between patients who received CBTp vs. other interventions at baseline. One-way ANOVA’s demonstrated that patients who received CBTp did not have significantly higher positive, negative, general or total psychotic symptoms at baseline than those who received other psychological interventions. Regression analyses showed no significant relationship between age, number of sessions, illness duration or any psychotic symptom measures. Crosstabs showed that gender, type of diagnosis, education level, occupation, and ethnicity were equally distributed between the intervention groups. Patients who received CBTp were significantly less often married (*11%*) and more often not married (*69%*) than patients who received other interventions (*16%* and 65% respectively, χ^2^ = 5.01, *p* = 0.03). The average number of sessions received significantly differed between patients who received CBTp (*M* = 14.75, *SD* = 5.78) and other interventions (*M* = 12.83, *SD* = 7.24, *F*(1) = 6.97, *p* = 0.01).

#### Efficacy of CBTp vs. Other Psychological Interventions

All results from the IPD meta-analyses examining the efficacy of CBTp vs. other psychological interventions are presented in **Table 2**. CBTp demonstrated superiority over other psychological interventions pooled at post-treatment for PANSS general symptoms (*b* = −0.17, *p* = 0.02), PANSS total symptoms (*b* = −0.15, *p* = 0.03) and when combining the total scores for the PANSS and BPRS across available RCTs (*b* = −0.16, *p* = 0.02). No significant difference was demonstrated for positive or negative symptoms.

**Table 2:** Individual participant data main effects of CBTp versus other interventions pooled.

Variable	Full sample of RCTs			RCTs assessed as low risk of bias		
	No of observations (no. of studies)	Mean (SE) βb	2-tailed *p* Value	No of observations (no. of studies)	Mean (SE) βb	2-tailed *p* Value
PANSS Positive symptoms	584 (11)	−0.10 (0.06)	.101	503 (8)	−0.13 (0.07)	.068
PANSS Negative symptoms	538 (10)	−0.69 (0.07)	.295	457 (7)	−0.05 (0.07)	.469
PANSS General symptoms	536 (10)	−0.17* (0.07)	.019	454 (7)	−0.08 (0.08)	.304
PANSS Total	538 (10)	−0.15* (0.07)	.027	456 (7)	−0.10 (0.08)	.168
BPRS Positive	119 (2)	−0.04 (0.16)	.823			
BPRS Negative	66 (1)	−0.02 (0.21)	.934			
BPRS Total	119 (2)	−0.16 (0.17)	.362			
SANS Total	143 (2)	−0.21 (0.14)	.135	90 (1)	−0.15 (0.17)	.380
Positive scales combined	703 (13)	−0.10 (0.06)	.114	503 (8)	−0.13 (0.07)	.068
Negative scales combined	747 (13)	−0.09 (0.06)	.110	547 (8)	−0.07 (0.70)	.297
Total scores combined	657 (12)	−0.16* (0.07)	.016	456 (7)	−0.10 (0.80)	.168

PANSS, Positive and Negative Syndrome Scale. BPRS, Brief Psychiatric Rating Scale. SANS, Scale for the Assessment of Negative Symptoms. RCT, Randomised Controlled Trial. SE, standard error. *p < .05.

#### Moderators of Psychotic Symptom Reduction in CBTp vs. Other Therapeutic Interventions

All IPD meta-analysis outcomes for sociodemographic and clinical variables as potential moderators of efficacy are presented in **Table 3**. Employment status significantly moderated the relationship between therapy type and combined negative psychotic symptoms at post-treatment when controlling for baseline negative psychotic symptoms. More specifically, patients who were students and received CBTp reported significantly lower negative psychotic symptoms at post-treatment than patients who were students and received other therapeutic interventions (*b* = −0.68, *p* = 0.04). To check whether this moderation could be explained by the age difference between the occupational groups, age was added as a covariate. The effect remained significant (*b* = −0.69, *p* = 0.04). On post- hoc examination of the effect, we determined a high likelihood of a chance finding due to very small numbers of students in the CBTp (*n* = 19) and ‘other psychological therapies’ group (*n* = 19) when compared to non-students (*n* = 253 in each the CBTp and other therapies groups) including instances of extreme outliers. We therefore excluded this comparison from further reporting in sensitivity analyses. No other significant moderators were found.

**Table 3:** Results of moderator analysis.

Moderator & psychotic symptoms outcome measure (z scores)	Full sample of RCTs			RCTs assessed as low risk of bias		
	*N* obs. (*N* stud.)	βb (*SE*)	*p*	*N* obs. (*N* stud.)	βb (*SE*)	*p*
Age						
Positive scales combined						
Treatment grp	699 (13)	0.04 (0.04)	.295	501 (8)	0.06 (0.05)	.265
Age × treatment grp		−0.01 (0.01)	.066		−0.01* (0.01)	.043
Negative scales combined						
Treatment grp	671 (13)	0.04 (0.04)	.310	473 (8)	0.03 (0.05)	.527
Age × treatment grp		−0.00 (0.01)	.789		0.00 (0.01)	.755
Total scores combined						
Treatment grp	653 (12)	0.07 (0.05)	.131	454 (7)	0.04 (0.05)	.480
Age × treatment grp		−0.01 (0.01)	.313		−0.00 (0.01)	.643
Gender						
Positive scales combined						
Treatment grp	703 (13)	0.07 (0.06)	.232	503 (8)	0.11 (0.07)	.104
Gender × treatment grp		0.06 (0.12)	.620		0.19 (0.14)	.187
Negative scales combined						
Treatment grp	747 (13)	0.12* (0.05)	.023	547 (8)	0.15* (0.07)	.020
Gender × treatment grp		0.06 (0.12)	.585		0.15 (0.13)	.275
Total scores combined						
Treatment grp	657 (12)	0.11 (0.06)	.085	456 (7)	0.10 (0.07)	.152
Gender × treatment grp		0.06 (0.13)	.624		0.19 (0.15)	.210
Education						
Positive scales combined						
Treatment grp	491 (9)	0.08 (0.07)	.208	293 (4)	0.17 (0.09)	.051
Tertiary vs secondary		−0.02 (0.15)	.876		0.30 (0.19)	.113
Negative scales combined						
Treatment grp	510 (10)	0.05 (0.06)	.447	312 (5)	0.05 (0.08)	.508
Tertiary vs secondary		−0.08 (0.14)	.565		−0.03 (0.17)	.879
Total scores combined						
Treatment grp	492 (9)	0.12 (0.07)	.084	293 (4)	0.13 (0.09)	.143
Tertiary vs secondary		−0.10 (0.15)	.522		0.18 (0.19)	.342
Marital status						
Positive scales combined						
Treatment grp	620 (11)	−0.04 (0.10)	.658	456 (7)	−0.03 (0.10)	.742
Not married vs married		−0.04 (0.17)	.830		−0.03 (0.18)	.863
Negative scales combined						
Treatment grp	621 (11)	−0.03 (0.10)	.734	457 (7)	−0.02 (0.10)	.857
Not married vs married		0.05 (0.17)	.742		0.13 (0.18)	.468
Total scores combined						
Treatment grp	621 (11)	−0.02 (0.10)	.843	456 (7)	−0.01 (0.10)	.920
Not married vs married		−0.05 (0.18)	.758		0.03 (0.18)	.857
Diagnosis						
Positive scales combined						
Treatment grp	636 (12)	0.06 (0.05)	.198	502 (8)	0.07 (05)	.167
Schizo-affective vs schizophrenia		−0.02 (0.21)	.918		0.11 (0.25)	.650
Other diagnosis vs schizophrenia		0.39 (0.25)	.115		0.04 (0.79)	.959
Negative scales combined						
Treatment grp	680 (12)	0.05 (0.05)	.256	546 (8)	0.04 (0.05)	.451
Schizo-affective vs schizophrenia		−0.08 (0.21)	.715		−0.19 (0.25)	.448
Other diagnosis vs schizophrenia		−0.19 (0.24)	.430		−0.13 (0.79)	.872
Total scores combined						
Treatment grp	590 (11)	0.08 (0.05)	.125	455 (7)	0.05 (0.06)	.358
Schizo-affective vs schizophrenia		0.19 (0.22)	.391		0.25 (0.25)	.332
Other diagnosis vs schizophrenia		0.13 (0.26)	.604		−0.71 (0.81)	.378
No. of sessions						
Positive scales combined						
Treatment grp	221 (6)	0.08 (0.08)	.345	141 (4)	0.16 (0.10)	.114
No. of sessions vs treatment grp		−0.01 (0.03)	.728		−0.03 (0.04)	.440
Negative scales combined						
Treatment grp	251 (6)	0.04 (0.08)	.634	171 (4)	−0.01 (0.09)	.886
No. of sessions vs treatment grp		−0.02 (0.02)	.438		−0.03 (0.02)	.213
Total scores combined						
Treatment grp	175 (5)	0.07 (0.10)	.465	94 (3)	0.08 (0.12)	.491
No. of sessions vs treatment grp		0.03 (0.04)	.421		−0.14* (0.06)	.024
Employment status						
Positive scales combined						
Treatment grp	509 (9)	0.07 (0.10)	.516	410 (6)	0.05 (0.11)	.639
Unemployed vs employed		0.04 (0.17)	.791		0.09 (0.18)	.636
Student vs employed		0.28 (0.34)	.416		0.17 (0.38)	.645
Negative scales combined						
Treatment grp	536 (10)	0.03 (0.10)	.772	437 (7)	0.07 (0.11)	.495
Unemployed vs employed		−0.06 (0.16)	.718		−0.06 (0.17)	.742
Student vs employed		−0.68* (0.33)	.039		−0.55 (0.37)	.135
Negative scales, controlling for age						
Treatment grp	526 (10)	−0.00 (0.10)	.992			
Unemployed vs employed		−0.07 (0.16)	.661			
Student vs employed		−0.69* (0.33)	.037			
Total scores combined						
Treatment grp	510 (9)	0.07 (0.11)	.539	410 (6)	0.08 (0.11)	.450
Unemployed vs employed		−0.01 (0.17)	.973		0.10 (0.18)	.573
Student vs employed		−0.35 (0.35)	.319		−0.17 (0.38)	.660
Ethnicity						
Positive scales combined						
Treatment grp	489 (8)	0.01 (0.80)	.949	328 (4)	0.14 (0.11)	.228
Other vs. Caucasian		−0.05 (0.15)	.721		0.11 (0.19)	.554
Negative scales combined						
Treatment grp	490 (8)	0.03 (0.08)	.678	329 (4)	0.10 (0.11)	.396
Other vs. Caucasian		−0.10 (0.15)	.489		0.07 (0.19)	.731
Total scores combined						
Treatment grp	490 (8)	−0.01 (0.09)	.946	328 (4)	0.13 (0.12)	.277
Other vs. Caucasian		−0.20 (0.16)	.206		0.01 (0.20)	.943
Illness duration						
Positive scales combined						
Treatment grp	383 (7)	0.03 (0.06)	.573	253 (3)	0.04 (0.07)	.627
Illness duration vs treatment grp		−0.01 (0.01)	.398		−0.01 (0.01)	.505
Negative scales combined						
Treatment grp	471 (8)	0.05 (0.05)	.282	341 (4)	0.04 (0.06)	.523
Illness duration vs treatment grp		−0.00 (0.01)	.663		−0.00 (0.01)	.960
Total scores combined						
Treatment grp	384 (7)	0.08 (0.06)	.207	253 (3)	0.03 (0.07)	.703
Illness duration vs treatment grp		−0.01 (0.01)	.395		0.00 (0.01)	.994
Baseline PANNS Severity						
PANSS Positive						
Treatment grp	537 (10)	0.04 (0.05)	.396	456 (7)	0.05 (0.05)	.353
PANNS Negative baseline severity vs treatment grp		0.01 (0.01)	.384		0.02 (0.01)	.178
PANSS Positive						
Treatment grp	537 (10)	0.04 (0.05)	.392	456 (7)	0.05 (0.05)	.347
PANNS General baseline severity vs treatment grp		0.01 (0.01)	.225		0.02 (0.01)	.098
PANSS Negative						
Treatment grp	538 (10)	0.04 (0.05)	.364	457 (7)	0.03 (0.05)	.522
PANNS General baseline severity vs treatment grp		−0.00 (0.01)	.832		0.01 (0.01)	.412

PANSS; Positive and Negative Syndromes Scale. RCTs; randomised controlled trials; SE; standard error.

#### Risk of Bias Sensitivity Analyses

Sensitivity analyses for risk of bias in the efficacy comparisons are presented in **Table 2**. Risk of bias sensitivity analyses on moderators are presented in **Table 3**. In the efficacy sensitivity analyses, the effects demonstrated previously were no longer significant for the PANSS general subscale (*b* = −0.08, *p* = 0.30), PANSS total symptoms (*b* = −0.10, *p* = 0.16) or the combined total scores of the PANSS and BPRS (*b* = −0.10, *p* = 0.17). Age was a significant moderator for combined positive symptoms; older patients who received CBTp reported significantly lower positive psychotic symptoms at post-treatment than younger patients who received other psychological interventions (*b* = −0.01, *p* = 0.04). Number of sessions was also found to be a significant moderator for total psychotic symptoms; patients who received CBTp and who received more sessions reported significantly lower total psychotic symptoms at post-treatment than patients who received less sessions and other psychological interventions (*b* = −0.14, *p* = 0.02). No other significant moderators were found.

#### Sensitivity Analyses on Conceptual Differences

Four studies included in the IPD used conceptually different aims and interventions than the remainder. Two used CBTp variants that were conceptually distinct; the first utilised individualised metacognitive training (MCT+. 24) a variant of CBTp targeting cognitive biases. Another utilised a cognitive–behavioural acceptance-based approach (36). Two studies were not primarily aimed at reducing psychotic symptoms therefore reported these as secondary outcomes (26, 27). We conducted additional sensitivity analyses in which all analyses were redone without these studies (**Supplementary Tables 4** and **5**). In the efficacy comparisons, CBTp demonstrated superiority over other psychological interventions at post-treatment for PANSS general symptoms (*b* = −0.19, *p* = 0.03) and for total psychotic symptoms as measured by the combined total scores of the PANSS and BPRS (*b* = −0.16, *p* = 0.04). No significant moderators were found.

#### Sensitivity Analyses on Treatment Format

Two RCTs utilised group rather than individual or mixed format (31, 40). Sensitivity analyses excluding these studies are presented in **Supplementary Tables 4** and **5**. CBTp demonstrated superiority over other psychological interventions for PANSS general symptoms (*b* = −0.18, *p* = 0.02), PANSS total symptoms (*b* = −0.17, *p* = 0.02) and when combining PANSS and BPRS total scores across RCTs (*b* = −0.16, *p* = 0.02). There were no significant moderators of treatment outcome.

## Discussion

To our knowledge, this is the first IPD meta-analysis examining the efficacy and moderators of psychological interventions for psychosis. Results were broadly consistent with conventional study-level meta-analyses research in demonstrating some superiority of CBTp over other psychological interventions although there was a slightly different pattern of results; CBTp was superior when combining any “total symptom” scores, on the PANSS total and on PANSS general symptoms. The previously observed effect on positive symptoms (3, 8) was not replicated using IPD. We note that including a smaller sample of RCTs due to failure to obtain databases for the whole eligible sample may have had impact; as a relative efficacy meta-analysis comparing bona fide interventions, power remained relatively low to detect small effects and prevent type 2 errors. The absence of superiority of CBTp for negative symptoms is consistent with our previous research (8, 9).

Our moderator analysis was exploratory based upon demographic and clinical variables available in the obtained databases. We found little evidence that any of these variables—age, gender, education level, marital status, diagnosis, employment status, ethnicity, illness duration or importantly baseline psychotic symptom severity—had significant impact upon treatment outcome. Sensitivity analyses and post-hoc examination demonstrated that the few significant moderating effects observed were not robust. This finding has clinical implications regarding assumptions about who may or may not benefit from psychological intervention; using demographic and clinical variables (e.g. severity of psychotic symptoms) in deciding whether or not a patient is allocated to psychological interventions may be unhelpful. This suggests that a broad range of patients with different backgrounds, circumstances, clinical presentations, symptom severity and clinical profiles may be equally able to benefit from psychological intervention. Our ability to reliably support this stance would be stronger with further development of our IPD database to include RCTs we were unable to obtain. This remains an important area of future research while adding absolute efficacy trials (versus treatment as usual) would also allow further insight.

Also of note was that patients who received a higher number of CBTp sessions had lower total psychotic symptoms at post-treatment than those who received less sessions and other therapies. This effect arrived *via* the sensitivity analysis minimising risk of bias which increases its validity. It is clinically acknowledged that severe mental health populations including psychosis patients are more likely to benefit from longer, more comprehensive interventions. However, this finding contrasts the beneficial effects reported in a meta-analysis of brief CBTp interventions, which also concluded that “dose” of sessions or contact time did not moderate treatment outcome (29). We note that conventional meta-analysis does not contain the facility to examine moderating effects at the individual participant level and therefore must rely on the less specific study-level data, such as mean number of sessions completed across participants. This therefore may provide less precise estimates. Our finding has implications for clinicians and service providers in suggesting that when investing in CBTp as opposed to minimal or supportive interventions, it is important that when feasible, a sufficient dose is provided rather than brief CBTp. Confirmation of this finding awaits future RCTs comparing conceptually-equivalent CBTp of varying length (e.g. 10 vs 20 sessions). We do not therefore intend this finding to act as justification to limit brief intervention in instances in which brief CBTp is the only viable option for specific services, risking further limitation in vital access to intervention.

We acknowledge various limitations. An inherent problem in IPD meta-analyses is availability bias due to difficulty obtaining RCT databases. We obtained 60% of eligible databases meaning that our IPD analyses did not include data from 40% of possible RCTs. Our conventional two-step meta-analysis did not suggest there were significant differences between included and non-included RCTs, although we are conscious of the possible impact that failure to obtain proportionately more eligible RCTs may have upon the power to detect effects despite improved precision using IPD. We encourage researchers to store data in a manner conducive to future collaboration and be open to database sharing since IPD may provide clearer insight for clinical decision-making than is possible with single RCTs or conventional meta-analysis.

A further limitation was the process subsuming variables into categories allowing meaningful inclusion in moderator analyses. Demographic and clinical variable availability, categorisation and reporting style varied across RCTs meaning we had the challenge of combining diverse information into broader categories. For example, marital status became “married” or “not married” since variation between databases meant it was not possible to reliably aggregate more nuanced data. This approach risks reductionism and limits the examination of differences between subgroups. We also note the inclusion of two RCTs of group-based CBTp (31, 40) and one RCT that combined group and individual approaches (21). While the inclusion of participant data from these RCTs was also at the individual level, the effects of group interventions may differ from those of individualised, case-formulation driven approaches. Our sensitivity analysis including only individual format RCTs (**Supplementary Materials**) demonstrated the same pattern of efficacy results as the main analysis. One strength is that all included RCTs utilised both blinding and intention-to-treat analyses, which improves the reliability of the results. Most RCTs also demonstrated minimal risk of bias.

This IPD meta-analysis suggested that CBTp is efficacious in reducing total and general symptoms of psychosis compared to other interventions. Results also suggested that patient characteristics, including psychotic symptom severity, do not significantly influence who benefits from these interventions. This finding has important implications for clinical policy and specifically for clinicians when deciding whether to refer or engage patients in therapy. Results also suggest that when investing in CBTp, the provision of a sufficient dose is important for treatment outcome. We note the exploratory nature of the findings from our moderator analysis.

## Data Availability Statement

The datasets analyzed in this article are not publicly available. Requests to access the datasets should be directed to d.t.turner@vu.nl.

## Author Contributions

DTT formulated the research objectives and designed the study, collected databases of existing trials, analysed data from these trials, wrote and edited the manuscript. MR helped design aspects of the study, formulated research questions, analysed data and wrote sections of the manuscript. MG formulated the research objectives and designed the study, facilitated the collection of existing trial databases, advised on data analysis and contributed to the development of all aspects of the manuscript. EK helped design aspects of the study, provided technical support on implementation of IPD technology, advised on data analysis and reviewed the manuscript. LV, SM, TL, DT, RP, HE, CC, FS, KO’C, Z-JL, and EB provided data from an original RCT contributing to the IPD sample and reviewed the manuscript drafts. PC formulated research objectives and designed the study, advised on data analysis, provided technical support on implementation of IPD technology and contributed to the development of the manuscript. Authors DTT, MR, MG, EK, and PC had access to the whole dataset. IPD co-authors (LV, SM, TL, DT, RP, HE, CC, FS, KO’C, EB, and Z-JL) provided datasets from original RCTs which they had full access to while access to the IPD dataset was available on request.

## Conflict of Interest

DT receives reimbursement for the provision of workshops on CBT for psychosis for the Insight-CBT Partnership, UK. Z-JL reports receiving a grant from Beijing Municipal Science & Technology Commission during the conduct of the RCT which he contributed to this IPD meta-analysis.

The remaining authors declare that the research was conducted in the absence of any commercial or financial relationships that could be construed as a potential conflict of interest.
