# Supplementary material for: Efficacy and Moderators of Cognitive Behavioural Therapy for Psychosis Versus Other Psychological Interventions: An Individual-Participant Data Meta-Analysis
Source: Front Psychiatry. 2020 May 5;11:402. doi: 10.3389/fpsyt.2020.00402 (PMC7214739; doi:10.3389/fpsyt.2020.00402)
Supplement: Supplementary file 1 [file DataSheet_1.docx]

**Supplementary materials**

**Supplementary Table 4.** *Individual participant data main effects sensitivity analyses*

|  | **Homogenous RCTs** |  |  |  | **Individual format** |  |  |
| --- | --- | --- | --- | --- | --- | --- | --- |
|  | No of observations (no. of studies) | Mean (SE) βb | 2-tailed *p* Value |  | No of observations (no. of studies) | Mean (SE) βb | 2-tailed *p* Value |
| **^Variable^** |  |  |  |  |  |  |  |
|  |  |  |  |  |  |  |  |
| PANSS Positive symptoms | 397 (7) | -0.06 (0.08) | .439 |  | 522 (10) | -0.11 (0.07) | .104 |
| PANSS Negative symptoms | 397 (7) | -0.04 (0.07) | .569 |  | 476 (9) | -0.08 (0.07) | .221 |
| PANSS General symptoms | 395 (7) | -0.19* (0.09) | .028 |  | 474 (9) | -0.18* (0.08) | .022 |
| PANSS Total | 398 (7) | -0.15 (0.08) | .065 |  | 476 (9) | -0.17* (0.07) | .021 |
| BPRS Positive | 119 (2) | -0.04 (0.16) | .823 |  | 53 (1) | 0.05 (0.25) | .837 |
| BPRS Negative | 66 (1) | -0.02 (0.21) | .934 |  |  |  |  |
| BPRS Total | 119 (2) | -0.16 (0.17) | .362 |  | 53 (1) | -0.10 (0.25) | .688 |
| SANS Total | 143 (2) | -0.21 (0.14) | .135 |  | 143 (2) | -0.21 (0.14) | .135 |
| Positive scales combined | 516 (9) | -0.06 (0.07) | .427 |  | 575 (11) | -0.10 (0.07) | .142 |
| Negative scales combined | 606 (10) | -0.08 (0.06) | .204 |  | 619 (11) | -0.11 (0.06) | .065 |
| Total scores combined | 517 (9) | -0.16* (0.07) | .037 |  | 529 (10) | -0.16* (0.07) | .023 |
|  |  |  |  |  |  |  |  |

**Supplementary Table 4**. PANSS, Positive and Negative Syndromes Scale. BPRS, Brief Psychiatric Rating Scale. SANS, Scale for the Assessment of Negative Symptoms. SE, standard error. RCTs, randomised controlled trials.

**Supplementary Table 5.** *Results sensitivity analyses for moderator analysis*

|  | **Homogenous RCTs** | | | **Individual format only** | | | |
| --- | --- | --- | --- | --- | --- | --- | --- |
|  | *N* observations (*N* studies) | Mean (SE) βb | 2-tailed *p* Value | *N* observations (*N* studies) | Mean (SE) βb | | 2-tailed *p* Value |
| **^Moderator & psychotic symptoms outcome measure (z scores)^** |  |  |  |  |  | |  |
|  |  |  |  |  |  | |  |
| Age |  |  |  |  |  | |  |
| Positive scales combined |  |  |  |  |  | |  |
| Treatment group | 512 (9) | 0.03(0.05) | .554 | 575 (11) | 0.04 (0.05) | | .355 |
| Age x treatment group |  | -0.01 (0.01) | .181 |  | -0.01 (0.01) | | .055 |
| Negative scales combined |  |  |  |  |  | |  |
| Treatment group | 530 (10) | 0.04 (0.05) | .465 | 547 (11) | 0.07 (0.05) | | .158 |
| Age x treatment group |  | -0.00 (0.01) | .753 |  | -0.00 (0.01) | | .952 |
| Total scores combined |  |  |  |  |  | |  |
| Treatment group | 513(9) | 0.07 (0.05) | .179 | 529 (10) | 0.07 (0.05) | | .146 |
| Age x treatment group |  | -0.01 (0.01) | .431 |  | -0.01 (0.01) | | .472 |
|  |  |  |  |  |  | |  |
| Gender |  |  |  |  |  | |  |
| Positive scales combined |  |  |  |  |  | |  |
| Treatment group | 516 (9) | 0.04 (0.07) | .563 | 575 (11) | 0.07 (0.06) | | .267 |
| Gender x treatment group |  | -0.01 (0.14) | .923 |  | 0.05 (0.13) | | .734 |
| Negative scales combined |  |  |  |  |  | |  |
| Treatment group | 606 (10) | 0.14* (0.06) | .026 | 619 (11) | 0.13* (0.06) | | .033 |
| Gender x treatment group |  | 0.06 (0.13) | .625 |  | 0.09 (0.13) | | .477 |
| Total scores combined |  |  |  |  |  | |  |
| Treatment group | 517 (9) | 0.11 (0.07) | .124 | 529 (10) | 0.10 (0.07) | | .157 |
| Gender x treatment group |  | 0.02 (0.15) | .902 |  | 0.05 (0.14) | | .715 |
|  |  |  |  |  |  | |  |
| Education |  |  |  |  |  | |  |
| Positive scales combined |  |  |  |  |  | |  |
| Treatment group | 451 (8) | 0.08 (0.07) | .233 | 427 (8) | 0.13 (0.07) | | .082 |
| Tertiary vs secondary |  | -0.05 (0.15) | .747 |  | 0.11 (0.16) | | .479 |
| Negative scales combined |  |  |  |  |  | |  |
| Treatment group | 470 (9) | 0.03 (0.07) | .654 | 446 (9) | 0.08 (0.07) | | .237 |
| Tertiary vs secondary |  | -0.12 (0.14) | .387 |  | -0.05 (0.15) | | .724 |
| Total scores combined |  |  |  |  |  | |  |
| Treatment group | 452 (8) | 0.11 (0.07) | .141 | 428 (8) | 0.16* (0.07) | | .031 |
| Tertiary vs secondary |  | -0.15 (0.16) | .362 |  | 0.05 (0.16) | | .763 |
|  |  |  |  |  |  | |  |
| Marital status |  |  |  |  |  | |  |
| Positive scales combined |  |  |  |  |  | |  |
| Treatment group | 480 (8) | -0.03 (0.10) | .742 | 495 (9) | -0.09 (0.12) | | .455 |
| Not married vs married |  | -0.06 (0.19) | .741 |  | -0.15 (0.21) | | .485 |
| Negative scales combined |  |  |  |  |  | |  |
| Treatment group | 480 (8) | -0.06 (0.10) | .556 | 496 (9) | -0.03 (0.11) | | .799 |
| Not married vs married |  | -0.04 (0.18) | .833 |  | -0.06 (0.20) | | .749 |
| Total scores combined |  |  |  |  |  | |  |
| Treatment group | 481 (8) | -0.05 (0.11) | .621 | 496 (9) | -0.10 (0.12) | | .440 |
| Not married vs married |  | -0.17 (0.19) | .389 |  | -0.21 (0.21) | | .323 |
|  |  |  |  |  |  | |  |
| Diagnosis |  |  |  |  |  | |  |
| Positive scales combined |  |  |  |  |  | |  |
| Treatment group | 449 (8) | 0.04 (0.06) | .468 | 575 (11) | 0.06 (0.05) | | .220 |
| Schizo-affective vs schizophrenia |  | -0.13 (0.25) | .602 |  | -0.11 (0.29) | | .696 |
| Other diagnosis vs schizophrenia |  | -0.38 (0.27) | .157 |  | 0.39 (0.25) | | .118 |
| Negative scales combined |  |  |  |  |  | |  |
| Treatment group | 539 (9) | 0.07 (0.05) | .188 | 619 (11) | | 0.05 (0.05) | .328 |
| Schizo-affective vs schizophrenia |  | 0.06 (0.24) | .789 |  | -0.12 (0.28) | | .653 |
| Other diagnosis vs schizophrenia |  | -0.20 (0.25) | .438 |  | -0.18 (0.24) | | .460 |
| Total scores combined |  |  |  |  |  | |  |
| Treatment group | 450 (8) | 0.09 (0.06) | .155 | 529 (10) | 0.08 (0.06) | | .151 |
| Schizo-affective vs schizophrenia |  | 0.12 (0.26) | .632 |  | 0.14 (0.30) | | .639 |
| Other diagnosis vs schizophrenia |  | 0.21 (0.27) | .437 |  | 0.14 (0.26) | | .593 |
|  |  |  |  |  |  | |  |
| No. of sessions |  |  |  |  |  | |  |
| Positive scales combined |  |  |  |  |  | |  |
| Treatment group | 134 (4) | 0.08 (0.12) | .467 | 221 (6) | 0.08 (0.08) | | .345 |
| No. sessions vs treatment group |  | -0.00 (0.04) | .989 |  | -0.01 (0.03) | | .728 |
| Negative scales combined |  |  |  |  |  | |  |
| Treatment group | 211 (5) | 0.03 (0.09) | .689 | 251 (6) | 0.04 (0.08) | | .634 |
| No. sessions vs treatment group |  | -0.01 (0.02) | .536 |  | -0.02 (0.02) | | .438 |
| Total scores combined |  |  |  |  |  | |  |
| Treatment group | 135 (4) | 0.04 (0.11) | .757 | 175 (5) | 0.07 (0.10) | | .465 |
| No. sessions vs treatment group |  | 0.04 (0.04) | .292 |  | 0.03 (0.04) | | .421 |
|  |  |  |  |  |  | |  |
| Employment status |  |  |  |  |  | |  |
| Positive scales combined |  |  |  |  |  | |  |
| Treatment group | 414 (7) | 0.06 (0.11) | .596 | 447 (8) | 0.04 (0.12) | | .739 |
| Unemployed vs employed |  | 0.04 (0.18) | .828 |  | -0.01 (0.19) | | .962 |
| Student vs employed |  | 0.29 (0.36) | .429 |  | 0.24 (0.35) | | .488 |
| Negative scales combined |  |  |  |  |  | |  |
| Treatment group | 440 (8) | 0.04 (0.10) | .706 | 474 (9) | -0.02 (0.11) | | .849 |
| Unemployed vs employed |  | -0.03 (0.16) | .879 |  | -0.12 (0.17) | | .485 |
| Student vs employed |  | -0.75* (0.35) | .030 |  | -0.71* (0.33) | | .033 |
| When controlling for age |  |  |  |  |  | |  |
| Treatment group | 430 (8) | -0.08 (0.16) | .614 | 466 (9) | -0.21 (0.17) | | .212 |
| Unemployed vs employed |  | -0.03 (0.17) | .854 |  | -0.11 (0.17) | | .540 |
| Student vs employed |  | -0.75* (0.35) | .030 |  | -0.68* (0.33) | | .042 |
| Total scores combined |  |  |  |  |  | |  |
| Treatment group | 415 (7) | 0.06 (0.11) | .557 | 448 (8) | 0.04 (0.12) | | .716 |
| Unemployed vs employed |  | -0.03 (0.18) | .874 |  | -0.07 (0.19) | | .706 |
| Student vs employed |  | -0.43 (0.37) | .241 |  | -0.38 (0.36) | | .281 |
|  |  |  |  |  |  | |  |
| Ethnicity |  |  |  |  |  | |  |
| Positive scales combined |  |  |  |  |  | |  |
| Treatment group | 393 (6) | -0.08 (0.10)- | .405 | 364 (6) | 0.03 (0.10) | | .751 |
| Other vs. Caucasian |  | 0.15 (0.17) | .432 |  | 0.07 (0.18) | | .709 |
| Negative scales combined |  |  |  |  |  | |  |
| Treatment group | 393 (6) | -0.01 (0.10) | .936 | 365 (6) | 0.08 (0.10) | | .432 |
| Other vs. Caucasian |  | -0.14 (0.17) | .421 |  | -0.11 (0.17) | | .519 |
| Total scores combined |  |  |  |  |  | |  |
| Treatment group | 394 (6) | -0.08 (0.10) | .422 | 365 (6) | 0.05 (0.11) | | .642 |
| Other vs. Caucasian |  | -0.27 (0.18) | .146 |  | -0.10 (0.19) | | .593 |
|  |  |  |  |  |  | |  |
| Illness duration |  |  |  |  |  | |  |
| Positive scales combined |  |  |  |  |  | |  |
| Treatment group | 383 (7) | 0.03 (0.06) | .573 | 383 (7) | 0.03 (0.06) | | .573 |
| Duration vs treatment group |  | -0.01 (0.01) | .398 |  | -0.01 (0.01) | | .398 |
| Negative scales combined |  |  |  |  |  | |  |
| Treatment group | 471 (8) | 0.05 (0.05) | .282 | 471 (8) | 0.05 (0.05) | | .282 |
| Duration vs treatment group |  | -0.00 (0.01) | .663 |  | -0.00 (0.01) | | .663 |
| Total scores combined |  |  |  |  |  | |  |
| Treatment group | 384 (7) | 0.08 (0.06) | .207 | 384 (7) | 0.08 (0.06) | | .207 |
| Duration vs treatment  group |  | -0.01 (0.01) | .395 |  | -0.01 (0.01) | | .395 |
|  |  |  |  |  |  | |  |
| Baseline PANNS Severity |  |  |  |  |  | |  |
| Positive scales combined |  |  |  |  |  | |  |
| Treatment group | 397 (7) | 0.04 (0.06) | .495 | 475 (9) | 0.04 (0.05) | | .413 |
| PANNS Negative baseline  severity vs treatment group |  | 0.00 (0.01) | .861 |  | 0.01 (0.01) | | .575 |
| Positive scales combined |  |  |  |  |  | |  |
| Treatment group | 397 (7) | -0.04 (0.06) | .502 | 475 (9) | 0.04 (0.05) | | .416 |
| PANNS General baseline  severity vs treatment group |  | 0.01 (0.01) | .507 |  | 0.01 (0.01) | | .165 |
| Negative scales combined |  |  |  |  |  | |  |
| Treatment group | 397 (7) | -0.03 (0.05) | .567 | 476 (9) | 0.05 (0.05) | | .277 |
| PANNS General baseline  severity vs treatment group |  | -0.01 (0.01) | .355 |  | -0.00 (0.01) | | .974 |
|  |  |  |  |  |  | |  |
|  |  |  |  |  |  | |  |

**Supplementary Table 5:** PANSS, Positive and Negative Syndromes Scale. RCTs, randomised controlled trials; SE, standard error.

**Table 6 (Supplementary): Adapted Cochrane Risk of Bias Tool**

| Study |  | Item 1 | Item 2 | Item 3 | Item 4 | Total risk |
| --- | --- | --- | --- | --- | --- | --- |
| Barretto *et al* 2009 |  | + | + | - | - | 2 |
| Cather *et al* 2005 |  | - | + | - | - | 1 |
| Durham *et al* 2003 |  | - | - | - | - | 0 |
| Garety *et al* 2008 |  | - | - | - | - | 0 |
| Haddock *et al* 2009 |  | - | - | - | - | 0 |
| Jackson *et al* 2008 |  | + | + | - | - | 2 |
| Lecomte *et al* 2008 |  | + | + | - | - | 2 |
| Li *et al* 2015 |  | - | - | - | - | 0 |
| Moritz *et al* 2011 |  | - | - | - | - | 0 |
| Penades *et al* 2006, 2010 |  | - | - | - | - | 0 |
| Penn *et al* 2009 |  | - | - | - | - | 0 |
| Sensky *et al* 2000, 2008 |  | - | - | - | - | 0 |
| Shawyer *et al* 2012 |  | - | - | - | - | 0 |
| Valmaggia *et al* 2005 |  | - | - | - | - | 0 |
|  |  |  |  |  |  |  |

**Table 6**. +, high risk of bias. -, low risk of bias. Item 1, random sequence generation. Item 2, allocation concealment. Item 3, blinding of assessors. Item 4, incomplete outcome data. Total risk of bias was calculated as the sum of high risk items to provide an overall risk score. Unclear risk of bias category was disregarded therefore when no information on an item was included in report, high risk of bias was assumed. All items were independently rated by two authors with conflicts resolved via discussion.

**Patient characteristics from RCTs included in IPD**

The 14 RCTs included a total of 898 patients. 460 received CBTp and 438 received other psychological interventions 539 (60%) were male. Eight of the 14 studies included only inpatients, while two included only outpatients. Four studies included inpatients and outpatients. Age ranged from 15 to 70 (mean 33.85, *SD* = 11.52). 342 patients (38%) completed secondary or lower education while 234 (26%) completed tertiary or higher education. Educational background status was unknown for four patients (0.5%) and not measured or missing for 318 (35.5%). 122 patients (13.6%) were married and 601 (66.9%) were not married. Marital status was unknown for one patient (0.1%) and not measured or missing for 174 (19.4%). 692 patients (77%) were diagnosed with schizophrenia, 72 (8%) with schizo-affective disorder, 51 (5.7%) with another psychotic disorder (schizophreniform disorder; bipolar/depression with psychotic features; delusional disorder; other psychosis or psychosis NOS) while diagnosis was not measured or missing for 83 patients (9.3%). The number of sessions ranged from 0 to 33 (*M* = 13.88, *SD* = 6.54). 152 patients (16.9%) were employed, 432 (48.1%) were not employed, 36 (4%) were students and employment was not measured or missing in 278 patients (31%). 247 patients (27.5%) were Caucasian and 327 (36.4%) identified as an another ethnicity (black/afro-Caribbean; Asian; Hispanic/Latin American; other). Ethnicity was not measured or missing in 324 patients (36.1%). Illness duration varied from 0 to 44 years (*M* = 9.57, *SD* = 8.56).

**Outcome measures overview**

Positive and Negative Syndrome Scale

The PANSS ^13^ is a 30-item clinician administered scale which provides a total score alongside subscales for positive, negative and general symptoms.

Brief Psychiatric Rating Scale

The BPRS ^14^ is a clinician-administered semi-structured scale primarily developed for assessing psychiatric symptoms, including psychotic symptoms. It provides an overall score and sub-categorisation into subscales including positive symptoms, negative symptoms, activation and affect. It exists as an 18-item or 24-item measure. ^15^

Scale for the Assessment of Negative Symptoms

The SANS ^16^ is a scale designed to assess the extent of negative symptomatology in psychosis. The SANS is divided into 5 subscales, namely affective flattening, alogia, avolition-apathy, anhedonia-asociality and attention which are rated 0-5 based on severity to provide a total score. We focused only upon total SANS score therefore did not analyse subscale data.

**Search strings:** Completed 25^th^ September 2017

**Medline/Pubmed**

Schizophrenia and Disorders with Psychotic Features AND (psychotherapy OR psychological intervention OR behaviour therapy OR cognitive therapy OR family therapy OR cognitive remediation OR social skills training OR sensory art therapies OR art therapy OR psychoeducation OR psychoanalytic therapy OR counseling OR supportive therapy)

-Limited to randomised controlled trials

**Result**: 2011 citations

**Psychinfo**

Schizophrenia OR psychosis AND (psychotherapy OR psychological intervention OR behaviour therapy OR cognitive therapy OR cognitive behaviour therapy OR family therapy OR family intervention OR cognitive remediation OR social skills training OR creative arts therapy OR psychoeducation OR psychodynamic psychotherapy OR counseling OR supportive therapy)

-Limited to clinical trials, Embase only (not Medline)

**Result**: 2457 citations

**Embase**

'Schizophrenia and Disorders with Psychotic Features' AND (psychotherapy OR psychological intervention OR behaviour therapy OR cognitive therapy OR family therapy OR cognitive remediation OR social adaptation OR art therapy OR psychoeducation OR psychoanalysis OR counseling OR supportive therapy)

-Limited to randomised controlled trials

**Result**: 1071 citations

**Cochrane Register**

Schizophrenia and Disorders with Psychotic Features AND (psychotherapy OR psychological intervention OR behaviour therapy OR cognitive therapy OR family therapy OR cognitive remediation OR social skills training OR sensory art therapies OR art therapy OR psychoeducation OR psychoanalytic therapy OR counseling OR supportive therapy) AND randomized controlled trial*

**Result**: 1498 citations

**Total from 4 databases**: 7037 citations

**After removal of duplicates:** 5881 citations

**List of eligible RCTs excluded due to failure to obtain data (8 RCTs)**

1. Bechdolf A, Knost B, Kuntermann C, Schiller S, Klosterkötter J, Hambrecht M, Pukrop R. Erratum: A randomized comparison of group cognitive-behavioural therapy and group psychoeducation in patients with schizophrenia (Acta Psychiatrica Scandinavica (2004) 110 (21-28)). *Acta Psychiatr Scand* (2004) 110:483. doi:10.1111/j.1600-0447.2004.00435.x

2. Drury V, Birchwood M, Cochrane R, MacMillan F. Cognitive therapy and recovery from acute psychosis: A controlled trial. I. Impact on psychotic symptoms. *Br J Psychiatry* (1996) 169:593–601. doi:10.1192/bjp.169.5.593

3. Klingberg S, Wölwer W, Engel C, Wittorf A, Herrlich J, Meisner C, Buchkremer G, Wiedemann G. Negative symptoms of schizophrenia as primary target of cognitive behavioral therapy: Results of the randomized clinical TONES study. *Schizophr Bull* (2011) 37: doi:10.1093/schbul/sbr073

4. Lewis S, Tarrier N, Haddock G, Bentall R, Kinderman P, Kingdon D, Siddle R, Drake R, Everitt J, Leadley K, et al. Randomised controlled trial of cognitive-behavioural therapy in early schizophrenia: Acute-phase outcomes. *Br J Psychiatry* (2002) 181: doi:10.1192/bjp.181.43.s91

5. Pinto A, La Pia S, Mennella R, Giorgio D, Sc D, Desimone L. Cognitive-Behavioral Therapy and Clozapine for Clients With Treatment-Refractory Schizophrenia. *Rehab Rounds.* (1999) 50:901–904.

6. Tarrier N, Barrowclough C, Vaughn C, Bamrah JS, Porceddu K, Watts S, Freeman H. The community management of schizophrenia. A controlled trial of a behavioural intervention with families to reduce relapse. *Br J Psychiatry* (1988) 153:532–542. doi:10.1192/bjp.153.4.532

7. Tarrier N, Beckett R, Harwood S, Baker A, Yusupoff L, Ugarteburu I. A trial of two cognitive-behavioural methods of treating drug-resistant residual psychotic symptoms in schizophrenic patients: I. Outcome. *Br J Psychiatry* (1993) 162:524–532. doi:10.1192/bjp.162.4.524

8. Tarrier N, Morrison AP, Hopkins R, Drake R, Lewis S, Haddock G. A pilot study evaluating the effectiveness of individual inpatient cognitive-behavioural therapy in early psychosis. *Soc Psychiatry Psychiatr Epidemiol* (1999) 34:254–258.
